# Acute myocardial infarction in young adults with Antiphospholipid syndrome: report of two cases and literature review

**DOI:** 10.4314/pamj.v8i1.71062

**Published:** 2011-02-22

**Authors:** Leila Abid, Faten Frikha, Zouhir Bahloul, Samir Kammoun

**Affiliations:** 1 Cardiology department, Hedi chaker hospital Sfax, Tunisia,; 2 Department of internal medicine, Hedi chaker hospital Sfax, Tunisia

**Keywords:** Antiphospholipid syndrome, acute myocardial infarction, coronarography

## Abstract

Abstract Acute myocardial infarction (AMI) is rarely associated with antiphospholipid syndrome. The treatment of these patients is a clinical challenge. We report the observations of 2 young adults (1 woman and 1 man), admitted in our acute care unit for acute myocardial infarction (AMI). A coagulopathy work-up concludes the existence of antiphospholipid syndrome (APS) in the 2 cases. APS syndrome was considered primary in 2 cases. All patients presented an intense inflammatory syndrome (high level of CRP). Anticardiolipine was present in the 2 cases. However, anti B2 glycoprotein I antibodies were detected in only one case. Emergency percutaneous transluminal coronary angioplasty (PTCA) with direct stenting had been performed successfully only in the first case, and the follow-up was uncomplicated. Thereafter, long-term oral anticoagulant appeared to be effective. The last patient was admitted because of peripheral acute ischemia of legs. Standard electrocardiogram showed signs of previous silent anteroseptal wall myocardial infarction confirmed by echocardiography. The latter revealed an apical thrombus and a very low left ventricular ejection fraction. Amputation of the right leg was necessary because of consultation occurred too late. However, he died four weeks later. Primary antiphospholipid syndrome should be considered as a cause of acute myocardial infarction in young adults, and PTCA with anticoagulant treatment is effective for initial treatment of this complication.

## Introduction

Antiphospholipid syndrome (APS) is an autoimmune disorder of acquired hypercoagulability characterized by the association of vascular thromboses (venous, arterial, small vessels) and or pregnancy morbidity (foetal loss, premature birth or recurrent embryonic losses) and persistent elevated serum levels of Antiphospholipid antibodies (anticardiolipin, lupus anticoagulant or anti- B2glycoprotein I) [1] It is also considered as a multisystemic disorder with a wide spectrum of presentations cutting across all subspecialties of medicine. Acute myocardial infarction (AMI) is rarely associated with this syndrome with a frequency of approximately 4%. The treatment of these patients is a clinical challenge. Herein, we report two cases of antiphospholipid syndrome in young adults revealed by acute myocardial infarction. Written informed consent was obtained from the patients for publication of this case report and accompanying images

## Patients and case reports 

**Case 1 **

**Figure 1:**
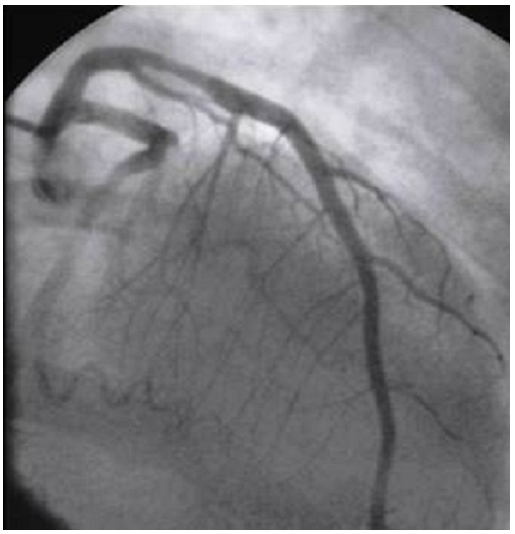
Left coronary arteriogram of a young patient with antiphospholipid syndrome and myocardial infraction on admission showing thrombotic stenosis of left descending coronary artery at the level of the proximal section

**Figure 2:**
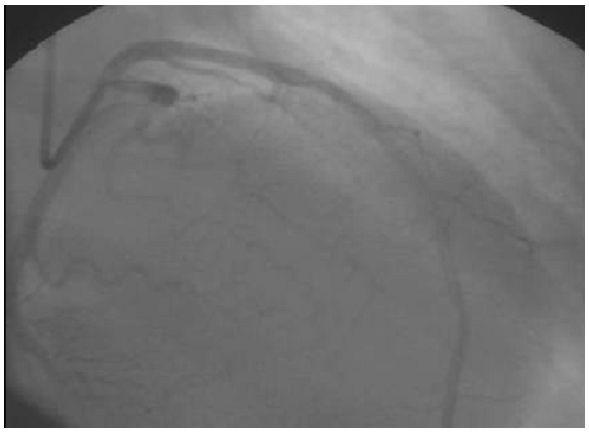
Arteriogram of a young patient with antiphospholipid syndrome and myocardial infraction after PTCA showing evidence of successful recanalization

A 30 years old woman with a history of recurrent spontaneous abortion was admitted with severe midsternal chest pain, associated with sweating, nausea, and breathlessness. She had no risk factors for atherosclerosis (diabetes mellitus, hyperlipidaemia, hypertension, and smoking). She had never previously experienced chest pain at rest or on exertion. During physical examination, she was pale and sweaty with a tachycardia of 120 beats/min. Her blood pressure was 80/40 mm Hg. There was no peripheral oedema and all peripheral pulses were present. She had a systolic apical murmur and symptoms of left heart failure. A 12 lead electrocardiogram revealed ST-segment elevation in leads V1, V2, V3, V4, V5, and V6. We diagnosed acute anterior myocardial infarction complicated by cardiogenic shock. Two-dimensional echocardiography revealed a markedly reduced left ventricular ejection fraction (30%) and akinesis of the anterior, lateral inferior wall, and inferior interventricular septum consistent with infarction in the left anterior descending area. Emergent coronary angiography was performed, the left anterior descending artery presented thrombosis and stenosis at the level of the proximal section (figure 1). Left ventriculography revealed that the left ventricle was dilated and markedly hypo kinetic. Therefore, percutaneous transluminal coronary angioplasty (PCTA) with direct stenting was performed, with successful recanalisation (figure 2). Serum creatine kinase peaked at 3500 U/l, haematological tests were normal, other laboratory tests of liver and renal functions and lipid profiles were normal. Anticoagulation by intravenous heparin infusion was performed for the next 10 days, Clopidogrel, aspirin, pravastatine were also administrated. Congestive heart failure was controlled by diuretics, nitrate, and low doses of dobutamine.

We performed haematological tests for thrombotic disorders. Blood platelet count was 119 000 /ml and prothrombin time was normal. Levels of protein S, C, ATIII were normal. Lupus anticoagulant was negative. ACL were searched by for enzyme-linked immunosorbent assays (ELISA). ACL levels were expressed in IgM and IgG units with positive levels > 20 Units (U). Anti-2GPI antibodies (IgG and IgM) were also detected by ELISA. Anti-2GPI antibodies and ACL were detected at positive levels. The levels of Anticardiolipin antibodies (Ig M antibodies) and anti-B2-glycoprotein titres were 23.41 MPL and 26.74 U/ml respectively.

There was no evidence for infection or any other triggering event before the MIs in this patient. Antinuclear antibodies and Anti DNA antibody were negative. Antiphospholipid antibodies tests repeated 12 weeks later remained positive. These findings satisfy the update Sapporo Classification criteria [1] for the diagnosis of antiphospholipid syndrome. This latter was considered primary because the patient had no signs of other systemic auto-immune diseases, particularly systemic lupus erythematosus (SLE). We decided to treat the patient with oral anticoagulation for life. The follow-up (24 months) was uncomplicated. Effort tests performed 3 and 6 months after PCTA were negative.

**Case 2 **

**Figure 3:**
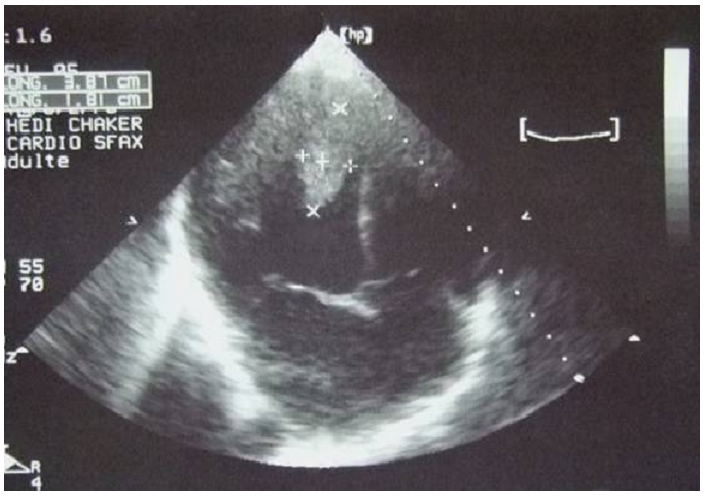
Four-chamber view echocardiography of a young patient with antiphospholipid syndrome and myocardial infraction showing an apical large thrombus measuring 38x18 mm

A 30-year-old Caucasian man was admitted in our intensive care unit because of bilateral acute ischemia of the two legs. He was regularly treated for Basedow disease. Smoking was the only risk factor of atherosclerosis disease. Physical examination showed a skinny patient. Peripheral pulses of the legs were absent. Arterial pressure was normal. The electrocardiogram showed a QS pattern in leads V1, V2, V3, and V4. Echocardiography revealed an apical large thrombus measuring 38 by 18 mm associated with a thinning left ventricular wall, suggesting painless myocardial infarction. It also demonstrated a markedly reduced left ventricular ejection fraction (19%) (Figure 3). The patient was immediately brought to the cardiovascular surgical department where bilateral Embolectomy to Fogarty probe was effectuated. Then amputation of the right leg was done because of so late consultation. Intravenous heparin was administrated with oral aspirin and clopidogrel.

Haematological tests showed normal levels of C, S and ATIII Protein. While FT4, FT3 were high, TSH was low. The patient was found to have a positive Lupus anticoagulant LA and a false positive VDRL. Anticardiolipin antibodies were also tested and subsequently came back positive for anticardiolipin antibody of the IgM isotype with a low level (19.25 MPL). The patient had no evidence for infection or any other triggering event before the MIs.

The patient was also found to have a false positive VDRL. Antinuclear antibodies, anti DNA, antiSm, anti SSA and anti SSB were negative. This was consistent with a primary antiphopholipid syndrome. Unfortunately, the patient died 15 days after his admission because of the failure of many of his organs (heart failure, acute renal failure, cytopenia).

## Discussion

The most common features of thrombotic disorders in antiphospholipid syndrome are deep vein thrombosis, pulmonary thromboembolism, and stroke. This syndrome is rarely initiated in the coronary arteries. On the other hand, acute myocardial infarction is unusual in young adults, but it has been reported in patients with antiphospholipid syndrome.

Antiphospholipid antibodies are the hallmark of the antiphospholipid syndrome which is characterized by thrombosis. It was first described in patients with systemic lupus erythmatosus and antiphospholipid antibodies [2] Antiphospholipid antibodies refer to auto antibodies that react to naturally occurring membrane bound phospholipids in human cells such as endothelial cells. These antibodies may be of IgG or IgM isotypes and consist of the lupus anticoagulant, anticardiolipin antibody, and false positive VDRL.

Antiphospholipid antibodies are associated with autoimmune diseases such as systemic lupus erythematosus, Patients often exhibit positive lupus anticoagulant activity but they infrequently suffer from the typical systemic lupus erythematosus (SLE) that satisfies diagnostic criteria. When they occur in isolation, this is known as primary antiphospholipid syndrome. The main antiphospholipid antibodies implicated in thrombosis and atherosclerosis are the anticardiolipin antibody, the lupus anticoagulant, and Ig G antibodies against plasma phospholipid –binding protein such as B2-glycoprotein I and prothrombin. Lupus anticoagulants were strong risk factors for both arterial and venous thrombosis and they are better predictors of thrombosis than anticardiolipin antibodies. Separate analysis of the different types of thrombosis showed anticardiolipin antibodies were associated with cerebral stroke and myocardial infarction but not with deep vein thrombosis.

Anticardiolipin antibody isotypes and titers are 2 important issues. Most studies investigated only IgG antibodies or did not distinguish between isotypes. With these limitations, IgM anticardiolipin antibodies were not associated with thrombosis, which limits their value in clinical practice. Anti-b2-glycoprotein I antibodies (anti-b2-GPI) represent one of the main subgroups of anticardiolipin antibodies (aCL) associated with antiphospholipid syndrome (APS). Positivity for anti-beta-2-glycoprotein I (anti-B2GPI) has been shown to be more closely associated with clinical manifestations of APS, including thrombosis.

Our two patients had both positive Ig M Anticardiolipin antibodies in a low level, but one had positive anti-B2-glycoprotein and the other had positive LA and so satisfied the criteria of primary APS. This can explain the occurrence of this coronary event.

Systemic arterial and venous thromboses are prominent and typical features of APS. Vianna et al [3] reported episodes of deep vein thrombosis in 54% and arterial occlusions in 44% of cases. The major cardiac manifestations of APS include valve disease, myocardial infarction, intracardiac thrombus and myocardial microthrombosis [4] The frequency of myocardial infarction in patients with APS is reportedly 4%, and the presence of antiphospholipid antibodies has been associated with myocardial infarction in young patients. In a multicenter prospective Cohort of 1000 patients with APS, MI was the presenting manifestation in 2.8 % and appeared during the follow-up in 5,5% of the Cohort [5] There is an increased risk of myocardial infarction in patients with APS [6] caused by coronary thrombosis rather than by premature atherosclerosis. In table 1 we summarise reports of cases of antiphospholipid syndrome with myocardial infarction since the report of Harris [7,11-13,15-25]The actual mechanism of the thrombosis in APS is as yet unknown, but numerous mechanisms have been proposed. It has been attributed to inhibition of prekallikrein by the lupus anticoagulant or, as recently postulated, to interference with the activation of the protein C and protein S anti-coagulant pathways. Recent data show that the biding of certain autoantibodies to endothelial cells and platelets is dependent upon the presence of B2 glycoprotein I (B2GPI). Autoantibodies binding to B2GPI on the surface of endothelial cells induce expression of adhesion molecules and enhance monocyte adhesion to the endothelial cells [8]

Current data are supporting an association between these autoantibodies and atherosclerosis as well. Human studies suggest that anti-cardiolipin antibodies and anti-beta2-glycoprotein-I antibodies are elevated in patients having coronary artery disease compared with controls [9] Anticardiolipin antibodies are also associated with typical chest pain, significant coronary artery stenosis on angiography and are predictive of myocardial infarction. Laboratory studies and murine models support the pro-atherogenic role of these autoantibodies, as they are involved in uptake of oxidized LDL into macrophages, and immunization of mice with them results in enhanced atherosclerosis [9] There is some evidence that high anti-beta2-glycoprotein-I antibodies can present a risk factor for atherosclerosis, but more epidemiological data are required in order to confirm whether the pro-atherogenic properties of anti-phospholipid antibodies signifies an independent risk factor for atherosclerosis and its complications. Hamsten et al [10] studied 62 patients who were survivors of acute myocardial infarction under the age of 45, and found that 21% of these patients had anticardiolipin antibodies. In those surviving with positive antibodies, 61% experienced additional cardiovascular events in the subsequent 5 years compared with those without elevated anticardiolipin antibodies. Mattila et al [11] demonstrated an increase in anticardiolipin antibodies in 52% of patients within three months of an acute myocardial infarction.

Thus, the interaction of these atherosclerotic and prothrombotic risk factors substantially increased the risk of myocardial infarction in the young patients. Subjects at risk of developing early coronary artery disease often have clustering of multiple risk factors (case 2).The optimal management of AMI in APS is still under debate [4] Primary angioplasty may be considered the treatment of choice in case of myocardial infarction. However, patients with APS have higher rates of complications after coronary angioplasty or CABG, such as early failure or recurrent coronary stent thrombosis [4] Anticoagulant therapy started immediately after the PTCA may contribute to cause long term coronary patency and to prevent acute stent occlusion. Therefore, long-term oral anticoagulant appeared to be effective. When thrombi are diffuse as in the second case, PTCA could not be done in emergency; intravenously platelet glycoprotein IIbIIIa receptor inhibitor may be effective. Fibrinolysis may be effective as initial treatment for acute thrombotic disorder including acute myocardial infarction (Harpaz et al and Ho et al) [12,13]

When patients presented catastrophic antiphospholipid syndrome with diffuse thromboembolism (like our second case), treatment with methylprednisone under a broad spectrum of anti-infective therapy may be effective. We didn’t practice it in our case. More aggressive therapy modalities may be necessary to save the patient’s life, such as plasmapheresis, immunoglobulin and cyclophosphamide.

For chronic management, the Committee consensus recommends aggressive treatment of all risk factors for atherosclerosis (hypertension, hypercholesterolaemia, and smoking) and liberal use of folic acid, B vitamins and cholesterol-lowering drugs (preferably statins) in patients suffering from APS. Hydroxychloroquine for cardiac protection in APS patients may be considered. The Committee also recommends warfarin anticoagulation for those who have a history of thrombosis in the absence of atherosclerosis, but it recognizes that developing data may support the use of antiplatelet agents instead. In presence of intracardiac thrombi, the Committee recommends intensive warfarin anticoagulation [14]; but in a recent study, high-intensity warfarin therapy (INR 3 to 4) was not superior to moderate-intensity warfarin for thromboprophylaxis in patients with APS and previous thrombosis [15]

## Conclusion

Acute myocardial infarction is unusual in young adults; antiphospholipid syndrome may be one of the aetiologies. Thus, immunological tests should be performed (lupus anticoagulant, anticardiolipin antibodies…), especially when there is a history of recurrent foetal loss or in the presence of symptoms of an associated connective tissue disorder. In such a case, PTCA followed by antithrombotic therapy is effective when angiographic data are favourable. Long term anticoagulant and antiplatelet therapies are recommended. Given the high rate of mortality of catastrophic antiphospholipid syndrome in some cases, this report emphasizes the need for rapid diagnosis and effective multimodal treatment in an intensive care unit setting for these patients.

## Competing interests 

The authors declare no competing interests.

## Author’s contribution 

ICMJE authorship criteria met. All the authors contribute to the management of the patient and the writing up of the manuscript. The final version of the manuscript was reviewed and approved by all the authors.

## Figures and Tables

**Table 1:** Literature data of some cases report of antiphospholipid syndrome with acute myocardial infarction

**Reference**	**Age**	**Sex**	**Lesion**	**Treatment**	**Other thrombosis**
Harpaz et al [11]	40	M	Anterior	t-PA (iv)	"Pulmonary embolism amaurosis fugax"
Kattwinkel et al [15]	29	F	Diffuse	Conventional medical treatment	Not described (recurrent fetal loss)"
Thorp et al [16]	29	F	Inferior	Conventional medical treatment"	DVT
Miller et al [17]	8	F	Lateral	Resuscitation death	Not described
Ho et al [12]	62	M	Anterior	t-PA (iv)	DVT
Sakakibara et al [18]	32	F	Inferior	Conservative CABG	Cerebral infarction
Chambers et al [19]	56	F	Inferior	Streptokinase (iv) PTCA CABG	Not described
Kovacs et al [20]	56	F	Diffuse	t-PA (iv)	DVT
Derksen et al [21]	32	F	Anterior	Conventional medical treatment	DVT
Susumu et al [22]	20	M	Inferior	PTCA t-PA (iv)	Not described
Stoupakis et al [23]	32	M	Anterior	PTCA	Not described
Ibrahim et al [24]	44	F	Inferior	Surgery (mass excision)	Cerebral infarction
Sajeev et al [25]	37	F	Anterior	Conventional medical treatment	Not described (fetal loss)
